# Lymphocyte Glucose and Glutamine Metabolism as Targets of the Anti-Inflammatory and Immunomodulatory Effects of Exercise

**DOI:** 10.1155/2014/326803

**Published:** 2014-06-02

**Authors:** Frederick Wasinski, Marcos F. Gregnani, Fábio H. Ornellas, Aline V. N. Bacurau, Niels O. Câmara, Ronaldo C. Araujo, Reury F. Bacurau

**Affiliations:** ^1^Department of Biophysics, Federal University of São Paulo, 04023-062 São Paulo, SP, Brazil; ^2^Division of Nephrology, Department of Medicine, Federal University of São Paulo, 04023-900 São Paulo, SP, Brazil; ^3^School of Arts, Sciences and Humanities, University of São Paulo, 03828-000 São Paulo, SP, Brazil; ^4^Department of Biodynamics of Human Movement, School of Physical Education and Sport, University of São Paulo, 05508-030 São Paulo, SP, Brazil; ^5^Department of Immunology, Laboratory of Transplantation Immunobiology, Institute of Biomedical Sciences, University of São Paulo, 05508-900 São Paulo, SP, Brazil

## Abstract

Glucose and glutamine are important energetic and biosynthetic nutrients for T and B lymphocytes. These cells consume both nutrients at high rates in a function-dependent manner. In other words, the pathways that control lymphocyte function and survival directly control the glucose and glutamine metabolic pathways. Therefore, lymphocytes in different functional states reprogram their glucose and glutamine metabolism to balance their requirement for ATP and macromolecule production. The tight association between metabolism and function in these cells was suggested to introduce the possibility of several pathologies resulting from the inability of lymphocytes to meet their nutrient demands under a given condition. In fact, disruptions in lymphocyte metabolism and function have been observed in different inflammatory, metabolic, and autoimmune pathologies. Regular physical exercise and physical activity offer protection against several chronic pathologies, and this benefit has been associated with the anti-inflammatory and immunomodulatory effects of exercise/physical activity. Chronic exercise induces changes in lymphocyte functionality and substrate metabolism. In the present review, we discuss whether the beneficial effects of exercise on lymphocyte function in health and disease are associated with modulation of the glucose and glutamine metabolic pathways.

## 1. Glucose and Glutamine Metabolism and Lymphocyte Function

Activated lymphocytes undergo a rapid burst in cellular proliferative, biosynthetic, and secretory activities and must obtain metabolic substrates to attempt this dramatic increase in metabolism [1]. Their insignificant intracellular store of nutrients obligates lymphocytes to markedly increase the uptake of metabolic substrates from their microenvironment [2]. Although lymphocytes are able to use glucose, glutamine, ketone bodies, and fatty acids (FA), it was determined that glucose and glutamine are quantitatively the most important fuel for activated lymphocytes [3].

Regarding the new metabolic demands of activated lymphocytes, glucose is initially retained in the cell by phosphorylation into glucose 6-phosphate by hexokinases (HKs) [2]. From there, glucose 6-phosphate can be used as a substrate by aerobic glycolysis or by the pentose-phosphate pathway (PPP). In the PPP, glucose 6-phosphate serves to generate ribose (for the synthesis of RNA and DNA) and NADPH (for FA synthesis) [1, 2].

For glucose 6-phosphate that enters aerobic glycolysis, the molecule is converted to pyruvate, after which it can be converted to lactate or acetyl-CoA or be fully oxidized [3]. The majority is converted to lactate (approximately 91%) [4–7], while most of the remaining pyruvate is converted to acetyl-CoA, which has a central role in membrane biogenesis [8], serving as a precursor to phospholipids, cholesterol, and triacylglycerol [1, 3]. Thus, only a small percentage of glucose 6-phosphate is fully oxidized in lymphocytes [3].

In this scenario, the removal of citrate (pyruvate converted to acetyl-CoA plus oxaloacetate) from the tricarboxylic acid (TCA) cycle for biosynthetic reactions imposes the need to continue replenishing intermediates to maintain this cycle's function [2, 9]. Thus, beyond glucose, activated lymphocytes also increase their update of glutamine and convert it to glutamate, which is in turn converted to *α*-ketoglutarate via glutamate dehydrogenase [3]. In addition to replenishing intermediates to maintain the TCA cycle using glutamate, lymphocytes also convert glutamine to aspartate and ammonia, providing biosynthetic precursors, purines, and pyrimidines, for the synthesis of DNA and RNA [3]. Finally, a limited percentage of glutamine can be converted to lactate or be fully oxidized [3]. In fact, although oxidative phosphorylation still occurs in effector T lymphocytes [8], it seems that, of the glutamine (and glucose) utilized by these cells, only approximately 1.5% is oxidized [3].

In accordance with the importance of glucose and glutamine in activated lymphocytes, early studies of these cells demonstrated that, to meet the new bioenergetic and biosynthetic demands imposed by activation, lymphocytes also increase the maximal activity levels of enzymes, such as HK, glucose-6-phosphate dehydrogenase (G6PDH) and phosphate-dependent glutaminase (GLUTase), which are key enzymes in the glycolysis, pentose-phosphate, and glutaminolysis pathways, respectively. The mitochondrial enzyme citrate synthase (CS), an important enzyme in the TCA cycle, is also affected [6, 7]. Comparatively, B cell metabolism has been less well investigated than T cell metabolism; however, the metabolic characteristics of both lymphocyte types might be similar [2, 10].

An outstanding feature of lymphocytes is that these cells utilize glucose and glutamine at high rates in a strictly function-dependent manner [7]. Furthermore, more recently, studies of direct modifications to T lymphocyte metabolic pathways demonstrated that metabolic reprogramming and lymphocyte activation are intricately linked, as cellular metabolism was found to be directly controlled by the signaling pathways that drive cell survival and activity [10]. Notably, Pearce and colleagues [8] stated that the reasons why T cells would adopt specific metabolic programs and the impacts of such programs on cell function and immunological responses were unclear.

In this sense, T lymphocytes adopt a metabolic program that reflects their energetic and biosynthetic needs in specific states, ranging from resting to memory cell conversion. For resting lymphocytes, it is worth mentioning that, despite the name, these cells continuously migrate through secondary lymphoid tissues to maintain immune surveillance prior to activation; to accomplish this activity, these cells rely on the oxidative metabolism of glucose, amino acids, and lipids [9]. However, as previously discussed, once activated, lymphocytes grow, proliferate, differentiate, and adapt to stress [8], and the mixed oxidative metabolism associated with a naïve resting state, which preferentially generates ATP, does not support these new functions [11]. Thus, the prioritization of the synthesis of macromolecules, rather than ATP production, explains the already-mentioned dependence of activated lymphocytes on aerobic glycolysis. In other words, the cell replaces its previous efficient ATP production (resting state) with efficient and rapid macromolecule biosynthesis (activated state) [11].

Additional support for the finding that cell metabolism is a key regulator of lymphocyte function and differentiation [11] has been provided by the patterns of “fuel usage” and transcriptional and posttranscriptional factors that control metabolism in the various activated T cell lineages. The helper T cell lineages Th1, Th2, and Th17 all exhibited increased aerobic glycolysis (as previously mentioned for activated lymphocytes), the posttranscriptional regulator mTORC1 was found to control glucose metabolism in Th1 and Th17 T cells, whereas mTORC2 controlled glucose in Th2 cells [9]. Th17 glucose metabolism is also controlled by the transcriptional regulator HIF-1*α* [9]. T regulatory (Treg) cells exhibit lipid oxidation as a primary metabolic phenotype, which is controlled by AMPK. Similarly, memory T cells also oxidize lipids, although in these cells, this metabolic phenotype is controlled by the posttranscriptional regulators TRAF6 and AMPK [11].

## 2. Lymphocyte Metabolic Dysregulation and Disease

The signals and stimuli that normally control the immune system (IS) can be affected by conditions such as obesity and type 2 diabetes (T2D) [12]. In this sense, it was proposed that the direct control of lymphocyte metabolism mediated by survival and activity-related signaling pathways could introduce the potential for metabolic changes to promote diseases [10]. More specifically, the inability of cell metabolism to meet the energetic and biosynthetic demands of lymphocytes could disrupt immune functionality, a process that has been observed in several immunological diseases [10].

For example, the inhibition of glycolytic metabolism can suppress cell proliferation and cytokine production and also compromise effector T cell differentiation [13]. In contrast, mitogen-induced T cell activation can reflect the glycemic statuses and insulin levels of type 1 diabetes and T2D patients [14]. Furthermore, hyperglycemia and ketoacidosis were found to increase the levels of proinflammatory cytokines and the numbers of activated T lymphocytes in diabetic patients [14].

Lymphocyte metabolic and/or functional dysregulation has been observed in a diet-induced obesity (DIO) model [15]. These phenomena were reported to promote reductions in the Treg and Th2 cell populations and increases in the resident inflammatory lymphocyte population [15]. Similarly, our laboratory recently reported that dendritic cells cultivated under leptin-free conditions exhibited a different phenotype from that of wild type cells; this phenotype was characterized by a reduced ability to induce CD4^+^ cell proliferation, while inducing increased Treg and Th17 cell differentiation [16]. These results proved useful for increasing our understanding of whether leptin can induce beneficial (increased Treg cell numbers) or detrimental (increased Th17 numbers) clinical outcomes [16].

In lymphocytes from Graves' disease patients, the maximal activity levels of HK, G6PDH, CS, and GLUTase were all reduced [17].* In vitro*, thyroid hormones were found to increase glucose and glutamine metabolism in the lymphocytes from these patients [17]. Additionally, it was reported that concomitant acute and chronic infections in patients with several diseases, such as cancer or asthma, are associated with an imbalance of T1 (T helper type 1 and T cytotoxic type 1) and T2 (T helper type 2 and T cytotoxic type 2) immune functions [18, 19].

In graft-versus-host disease (GVHD), T lymphocytes are activated within a systemic inflammatory environment containing ubiquitous antigens [20]. One of the few studies that investigated T lymphocyte metabolism* in vivo* demonstrated that cells activated under GVHD conditions became highly dependent on lipid metabolism, rather than exhibiting the expected increase in glycolytic metabolism [20]. In accordance with the results of that study, the FA metabolism dependence of allogeneic T cells distinguishes these cells metabolically from other activated T cell subsets, thus providing targets for therapeutic intervention (e.g., blockade of FA transport, inhibition of FA oxidation, and limitation of fuel sources) [20].

Cancer is another condition associated with inflammation. In fact, the concept that an inflammatory tissue injury could induce neoplasia and the existence of a close relationship between carcinogenesis and inflammation were initially postulated by the Greek physician Galenus approximately 2000 years ago [21]. Current estimations suggest that approximately 25% of cancers require a chronic inflammatory microenvironment for development [21]. Additionally, obesity, which is associated with chronic low-grade inflammation, increases the risk of developing certain types of cancers [22].

Therefore, in accordance with the hypothesis suggested by Caro-Maldonato and coworkers [10], lymphocytes from animals and humans with cancer are expected to exhibit metabolic dysregulation or mismatches. In fact, cells from primary effusion lymphoma (PEL), a subtype of B cell non-Hodgkin's lymphoma with a median patient survival duration of 6 months, provide support for this hypothesis [23].* In vitro*, these cells exhibit an increased dependence on aerobic glycolysis through the PI3 K/AKT/mTOR pathways that control glycolysis via GLUT1 [24]. Additionally, PEL cells actively convert glucose to FAs via increased FA synthase activity. In fact, these cells, as well as those from other B cell non-Hodgkin's lymphoma subtypes, are dependent on FA synthase to such an extent that Bhatt and coworkers [23] suggested the possibility of using this enzyme as a unique molecular treatment target in these cancers.

Three possible outcomes of the occurrence of a mismatch or insufficient fuel usage in T lymphocytes were described [10]. The first was altered or inhibited Th1, Th2, and Th17 differentiation. The second was the inhibition of proliferation or induction of cellular senescence. The third was the induction of cell death. In accordance with these results, the above-described outcomes for T and B lymphocytes have been observed in several immunological diseases and have also provided opportunities to selectively modulate specific immune functions by targeting glucose, lipid, and amino acid metabolism [10].

## 3. Mediators of Exercise-Induced Immunomodulation

### 3.1. Catecholamines and Cortisol

The studies mentioned in this section were designed in accordance with two main research approaches that have been proposed to establish a link between exercise and the IS: a metabolic approach, which considers plasma glutamine concentrations/metabolism, and a neuroendocrine approach, which considers changes in the levels of immunomodulatory hormones and neurotransmitters [25].

Interestingly,* in vitro* studies demonstrated that the “stress hormones” adrenaline and cortisol are able to modulate lymphocyte metabolism. The organism exerts tight control over the internal environment, and any subtle disruption of the regulated limits triggers physiological feedback mechanisms to reestablish the internal milieu [26]. Among these mechanisms is integration of the nervous and endocrine systems (or the neuroendocrine system, NES) with IS [27], which is systematically controlled by the NES [26]. Of note, the integrated communication between the NES and the IS possibly consists of the sharing of common signaling proteins and their corresponding receptors [28, 29].

In the face of any stimuli able to disrupt homeostasis, the response of the NES is invariably (regardless of the nature of the stress) the same: activation of the sympathoadrenal (SA) system and consequent release of catecholamines (i.e., adrenaline and noradrenaline) and activation of the hypothalamic-pituitary-adrenal (HPA) axis, which, in humans, results in cortisol production and release [30, 31].

The stereotypic response of the NES to stress [32] aids in understanding of the effects of exercise upon the IS because among the several molecules (e.g., hormones, cytokines) able to affect immune cell function, catecholamines, and cortisol appear to be particularly involved in exercise-induced immune responses [33]. Specifically, activation of the SA system occurs several seconds after exercise initiation, whereas the HPA response and the secretion of cortisol often need 20–30 minutes before beginning [34]. Additionally, catecholamines appear to be responsible for the initial effects of acute exercise on the IS (e.g., the migration of lymphocyte subpopulations) [35], whereas cortisol appears to exert its effects within a period of at least 2 hours [35].

An interesting feature of exercise is that in accordance with Fragala et al.'s [36] work exercise presents a unique stress on the homeostatic conditions, and this stress is specific to the nature and configurations of the protocol and associated elements (i.e., the environmental conditions or nutritional status). That is, the magnitude of NES activation in response to exercise stress is determined by the intensity and duration of such exercise [37]. Consequently, the exercise protocols that most affect the IS are those in which the intensity and duration (acute variables) and frequency (chronic variable) are higher. Not surprisingly, aerobic exercise protocols with longer durations (>1.5 hours) and greater intensities (55/60–75% of VO_2_max) induce greater release of catecholamines and cortisol in comparison with aerobic exercise at lower intensity [38].

Discussing the effects of different intensities, durations, and modes of exercise on the response of the NES is obviously beyond the scope of this review. However, briefly, noradrenaline presents a curvilinear increase in response to acute exercise as workload augments, while adrenaline increases at workloads over 60% VO_2_max [39]. Regarding cortisol, mild-intensity, moderate-duration aerobic exercise does not appear to alter its levels. However, exercises with intensity above 85% VO_2_max [40, 41] or with a duration greater than 60 minutes [42] typically lead to increases in cortisol secretion.

Rosa and coworkers [43] previously demonstrated the ability of adrenaline to increase the proliferative index of mesenteric lymphocytes and to concomitantly augment the maximal activities of HK, GLUTase, and CS, as well as glucose and glutamine consumption. However, the excess of systemic catecholamines induced by high-intensity, exhaustive exercise could have an immunosuppressive effect, such as a reduction in the plasma levels of interferon-*α* (IFN*α*) and an antiviral cytokine [44]. In addition to its antiviral property, IFN*α* has antiapoptotic and antiproliferative effects on activated lymphocytes [45]. Regarding the effects of this cytokine on lymphocyte metabolism, we demonstrated that the ability of IFN*α* to limit T and B cell proliferation could be explained by the suppression of glucose and glutamine metabolism and reduced maximal G6PDH, CS, and GLUTase activities [46].

The immunosuppressive effect of glucocorticoids on mesenteric lymphocytes is associated with a 40% reduction in pyruvate utilization due to inhibition of pyruvate dehydrogenase's maximal activity [47].

Additionally, as mentioned, cortisol increases are typically observed during high-intensity exercises. We recently demonstrated that basketball players participating in an official game (stressful exercise) presented increases in salivary cortisol and a reduction in levels of interleukin (IL)-21, a cytokine that stimulates immunoglobulin A-secreting cells [48]. Additionally, in accordance with the immunosuppressive ability of cortisol, it was observed that the numbers and proliferative ability of circulating lymphocytes are affected by a single bout of intense exercise [38]. For example, a 30–50% decrease in the lymphocyte count occurs at 30 minutes after exercise [38].

Regarding the proliferative index of lymphocytes, our results demonstrated that participation in very intense exercise protocols, an Olympic triathlon (swimming for 1.5 km, cycling for 40 km, and running for 10 km) [49, 50] or a simulated cycling competition (6 sets of 20 minutes at 90% of the individual's anaerobic threshold) [51] reduced the proliferative indices of T and B lymphocytes and plasma glutamine levels. These changes were associated with reduced cytokine production (e.g., IL-1, IL-2, IL-4, tumor necrosis factor (TNF)*α*, and interferon (IFN)*γ*) by mononuclear peripheral cells in response to mitogens. It was proposed that the IFN*γ*/IL-4 ratio in the culture supernatants of stimulated T cells could act as an objective indicator for monitoring the Th1/Th2 balance (cell-mediated/humoral immunity) [19]. As such, these studies suggested that an intense bout of acute exercise could affect this balance [19, 49–51].

To maintain homeostasis in the face of all organic changes induced by physical exercise (e.g., increased body temperature, dehydration, ion imbalances, hypoxia, and blood pressure changes), other hormones (e.g., insulin, growth hormone, aldosterone, glucagon, and thyroxin) are necessary in addition to cortisol and catecholamines during exercise and recovery [32]. Although the involvement of these hormones in exercise's ability to modulate the IS needs further investigation, certain evidence reinforces possible involvement. For example, insulin can control the metabolism and functionality of lymphocytes [1], and its infusion into critically ill patients is used to achieve tight hyperglycemic control and to fight systemic inflammation [52]. Thyroid hormones in turn stimulate aerobic glycolysis, glutamine consumption, and aerobic metabolism in human lymphocytes [17].

### 3.2. Cytokines

As previously discussed regarding the effect of an excess of catecholamines on plasma TNF*α* [44], the plasma levels of other cytokines are affected by exercise [27], and it has been suggested that the impact of exercise on cytokine production could partially explain how this stressor modulates the IS [32]. Regarding this concept, the cytokine that is most responsive to exercise is IL-6, whose levels increase up to 100 fold as the duration of exercise progresses [53]. Interestingly, the source of this cytokine is the skeletal muscle [54]. The increase in IL-6 levels is followed by an increase in the levels of an IL-1 receptor antagonist that is an inhibitor of the inflammatory cytokines IL-1 and IL-10, important anti-inflammatory cytokines [22].

### 3.3. Other Mediators

In addition to the neuroendocrine and immune responses to exercise mentioned above, exercise may modulate immunity in alternative ways. One of these ways is exercise-induced muscle damage, which results in the secretion of inflammatory cytokines by innate immune cells [33]. Another way is the effect of exercise upon plasma glutamine levels [55]. Thus, the reduction in the levels of this amino acid after strenuous exercise could be related to immunosuppression, whereas the increase in glutamine levels induced by chronic moderate-intensity exercise would induce a positive effect upon immune function [56].

Finally, it is worth mentioning that, in many respects, the responses and adaptations to chronic exercise are the result of the cumulative influence of repeated acute exercise bouts [57]. Thus, to understand how exercise training can modulate lymphocyte function as well as metabolism, it is important to know the acute effects of exercise.

## 4. Anti-Inflammatory and Immunomodulatory Effects of Chronic Exercise

Regarding the chronic effects of physical exercise, the protection that it offers against all-cause mortality is known to occur primarily due to the ability of exercise to protect against atherosclerosis, T2D, colon cancer, and breast cancer [22]. The fact that the above-mentioned diseases seem to associate with low-grade chronic inflammation and the finding that acute exercise increases the systemic levels of several anti-inflammatory cytokines (especially exercise that is not high intensity or exhaustive) suggest the possibility that regular exercise can protect against the chronic conditions associated with low-grade inflammation via an anti-inflammatory effect [22].

In accordance with certain authors, the potential underlying mechanisms of the anti-inflammatory effects of regular moderate exercise primarily include a reduction in visceral fat mass, increased production, and release of myokines and reduced expression of Toll-like receptors on monocytes and macrophages; however, these mechanisms likely also include inhibition of monocyte and macrophage infiltration into adipose tissues, a reduction in the number of circulating proinflammatory monocytes and an increase in the number of circulating Treg cells [58]. The modulation of lymphocyte metabolism by exercise, however, is not included on this list.

In accordance with the proposal that regular moderate-intensity exercise has beneficial anti-inflammatory (and immunomodulatory) effects, it seems that when chronic, this exercise intensity is able to reverse the age-associated reduction in Th1 cell numbers or Th1-cell derived cytokine levels that are normally observed in older adults [19]. Moreover, other studies have reported that moderate exercise training increased the production of Th1 response-associated cytokines in both humans and rats [59–63]. Still, experimental evidence suggests that chronic moderate exercise could normalize IL-4 concentrations and increase IL-2 concentrations in a heart failure model in which the T2-type response had been initially elevated [64].

The evidence suggests that high-intensity exercise could be immunosuppressive [12]. It was demonstrated that when chronic, this level of exercise stimulates a type 2 T cell phenotype in trained individuals [19] and that this tendency might be associated with the high incidence of upper respiratory infection episodes among athletes [12]. In fact, two recent studies have suggested that lifelong participation in high volumes of intense exercise could compromise immune functionality [65, 66]. Together, these studies reported that young athletes had fewer CD4^+^ T lymphocytes and that these cells exhibited reduced functionality and a higher degree of differentiation in comparison with those from young nonathletes. Regarding CD8^+^ T lymphocytes, it was observed that despite, the higher number of these cells, there was a lower frequency of thymic emigration [65]. However, this altered lymphocyte functionality observed in young athletes was not found in elderly athletes; however, in the latter group, natural killer cells exhibited increased activation and degranulation. Therefore, it is possible that, in elderly individuals, the IS can adapt itself to the detrimental effects of lifelong exhaustive exercise [65].

Together, these studies suggest that chronic exercise affects lymphocyte functioning. Thus, considering the strict relationship between function and metabolism in these cells, it is important to understand how glucose and glutamine consumption in lymphocytes is modulated by regular moderate- and high-intensity exercise.

## 5. Chronic Exercise-Mediated Modulation of Lymphocyte Nutrient Metabolism

Evidence suggests that these effects of exercise are at least partly due to the ability of exercise to modulate cell nutrient metabolism and particularly glucose and glutamine metabolism.

For example, Navarro and colleagues [67] demonstrated the ability of chronic moderate-intensity exercise (eight weeks of treadmill running) to modulate the activation, proliferation, cytokine production, and glucose and glutamine metabolism of T and B lymphocytes.

In support of the statement that lymphocytes utilize glucose and glutamine at high rates according to their specific immune functions [68], the exercise-induced metabolic changes observed by Navarro and colleagues [45] were accompanied by concomitant alterations in functionality. For example, increased expression of IL-2 and its receptor (IL-2R) and decreased expression of IL-4 and its receptor (IL-4R) were observed in T cells relative to B lymphocytes. These data suggest that chronic moderate exercise in healthy animals primarily enhances the Th1 response phenotype [67]. As several immunological disorders have been associated with a dysregulated Th1/Th2 balance [69], this is an important finding.

Regarding humoral immunity, chronic moderate exercise was shown to increase IgG production in lymphocytes from trained rats compared with lymphocytes from sedentary animals, thus indicating an improvement in humoral immunity. In support of the finding that both cellular and humoral immune functions improved in response to exercise, increases in the expression and modulation of CD8, CD54, and CD30 were observed, potentially indicating improvements in both types of immunity [67].

It was observed that the changes in lymphocyte function were accompanied by a differential effect of moderate exercise on T and B lymphocyte metabolism [67]. Specifically, T lymphocytes increased glutamine utilization by shifting the metabolism of this amino acid to an aerobic pathway (as previously mentioned herein, only a minor percentage of glutamine is oxidized in lymphocytes). Concomitantly, these cells reduced their glucose consumption and lactate production levels (lymphocytes typically convert most of their glucose to lactate). In contrast, B lymphocytes exhibited increases in both glucose and glutamine consumption, although only glutamine aerobic metabolism was increased [67]. All of these lymphocytic changes were possible because key glucose and glutamine metabolic enzymes were targets of the modulatory effect of chronic exercise. Therefore, in accordance with enhanced aerobic glutamine metabolism, the maximal activities of GLUTase and CS increased in T lymphocytes' response to exercise. In addition to these 2 enzymes, the maximal activities of HK and G6PDH were also augmented in B lymphocytes in response to chronic exercise [67].

The effects of chronic moderate-intensity exercise were also investigated in animal models of chronic diseases. Recently, activated T lymphocytes were proposed as a model to understand carcinogenesis [70]. Through changes in transporter expression and isozyme switching, both activated lymphocytes and cancer cells become highly glycolytic and glutamine dependent to promote growth, proliferation, and differentiation. Therefore, an understanding of the metabolism of activated T cells could facilitate the identification of new therapeutic strategies that would selectively target tumor metabolism or inflammatory immune responses [70].

Thus, the effects of exercise were investigated in lymphocytes obtained from Walker-256 tumor-bearing rats [71]. In response to the tumor, the metabolism and function of these cells were compromised; T and B cells from the tumor-bearing rats exhibited lower proliferative indices relative to those of cells from sedentary animals [71] and increased glucose consumption and lactate production in comparison with cells from control animals. Eight weeks of moderate-intensity treadmill running suppressed tumor growth and reversed the repressive effects of the Walker-256 tumors on the lymphocytes' proliferative indices. Additionally, exercise training reversed the effect of the tumor by reducing glucose consumption and lactate production while counterbalancing the effects of the disease on the maximal activities of G6PDH, HK, and CS. Therefore, the immunomodulatory effects of exercise were characterized by a reversion of the tumor-induced changes. Finally, the exercise-induced effects on lymphocyte function and metabolism were accompanied by altered plasma hormone levels (e.g., growth hormone, testosterone, and corticosterone) and beneficial changes in cytokine levels (e.g., IL-1, IL-2) [71].

Rheumatoid arthritis (RA) is an autoimmune disease that causes several disturbances in immunological functioning [72, 73]. Previously, it had been speculated that because certain immune functions are exacerbated in RA, improved IS functioning with exercise could theoretically be detrimental to RA patients. However, the opposite was found to be true, as studies performed in the 1990s regarding exercise and inflammatory disease demonstrated that nearly any type of exercise was superior to a sedentary lifestyle for RA patients [74, 75]. Additionally, it was observed that exercise appeared to be beneficial for individuals with RA because of its anti-inflammatory effects [22, 74].

**Figure 1:**
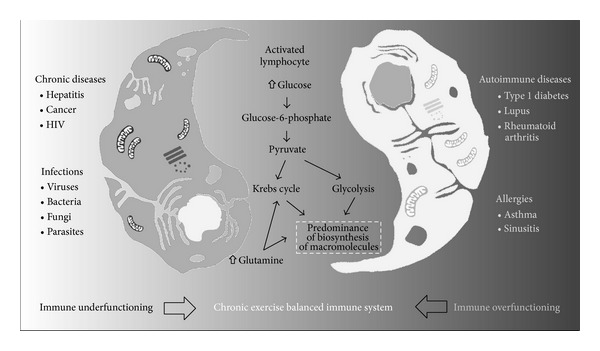
Exercise counterbalances lymphocyte metabolic dysregulation, modulating several components of glycolysis and glutaminolysis. HIV—human immunodeficiency virus.

Therefore, we decided to verify whether alterations in glucose and glutamine metabolism were present in a model of experimental arthritis (collagen-induced arthritis, CIA) and whether a chronic swim training regimen could counterbalance the deleterious effects of RA by modulating the metabolism of these nutrients [62]. Initially, we observed that lymphocytes from CIA animals consumed more glucose, despite exhibiting reduced lactate production relative to lymphocytes from healthy animals, thus indicating that CIA induced “defective” lymphocyte activation [62]. Additionally, CIA reduced glutamine consumption and glutamate/aspartate production, and these metabolic changes were associated with an elevated proliferative index in the cells from CIA animals. However, an eight-week moderate-intensity swim training regimen reduced the proliferative index and glucose consumption of lymphocytes from the CIA animals and increased their glutamine metabolism (Figure 1).

To obtain a more complete understanding of the immunomodulatory effects of exercise, we also analyzed the plasma levels of certain hormones. Trained CIA rats exhibited lower levels of the proinflammatory hormone prolactin and higher levels of the immunosuppressive hormones progesterone and corticosterone [62]. Interestingly, chronic exercise also increased the plasma levels of IL-2, a cytokine that can both initiate and terminate inflammation under different conditions, as well as increased plasma levels of glutamine [76]. Taken together, the data from the trained CIA animals suggested that the ability of swim training to counterbalance several effects of CIA resulted from the modulation of lymphocyte metabolism and the balance between proinflammatory and anti-inflammatory hormones and cytokines.

Despite the common belief that high-intensity exercise is immunosuppressive [12], it is important to note that, in this case, “high intensity” means chronic exercise of high intensity and volume, such as those physical training regimens used by athletes [12]. That is, it is possible to speculate that high-intensity exercise at a low/moderate volume would induce beneficial effects on the IS because this type of exercise would allow the organism to become adapted to it.

In support of this speculation, an eight-week anaerobic jumping training (a high-intensity exercise) regimen was reported to increase the expression of the proapoptotic protein Bax and reduce the expression of the antiapoptotic protein Bcl-2; these findings were associated with reduced Walker-256 tumor growth consequent to apoptosis [77]. The authors attributed their findings to unknown interference in the Walker-256 tumor cells. Subsequently, our group observed that Walker-256 tumor-bearing rats subjected to high-intensity running training for an 8-week period (85% VO_2_max thirty minutes per day for five days) exhibited a 40% reduction in tumor growth and a 35.5% increase in lifespan relative to sedentary, tumor-bearing rats [63]. These changes were accompanied by reduced lactate production in the Walker-256 tumor cells, suggesting that the tumor cells had become less glycolytic. Moreover, in the trained animals, the tumor cells exhibited increased glutamine consumption and glutamate and aspartate production. Despite these findings, glutamine consumption due to aerobic metabolism was reduced [63].

Regarding lymphocyte function and metabolism, the same study demonstrated that the exercise training protocol counterbalanced the effects of the Walker-256 tumors on lymphocyte metabolism [63]. Lymphocytes from trained animals exhibited an increased proliferative index, reduced glucose consumption (aerobic and anaerobic), and reduced lactate production in comparison with immune cells from sedentary, tumor-bearing rats. Similarly, the high-intensity exercise program reversed the tumor-induced effects on glutamine metabolism [63]. Notably, the modulatory effect of exercise was accompanied by increased cytokine levels (e.g., IL-1, IL-2, and TNF*α*) and changes in plasma hormone levels (e.g., increased corticosterone levels, reduced growth hormone levels) [63]. Therefore, the immunomodulatory effects of exercise occurred in response to a complex interaction of hormones, cytokines, and metabolic changes.

## 6. Conclusion

Acute exercise and chronic exercise affect lymphocyte function in a manner associated with the modulation of glucose and glutamine metabolism. Although further studies are necessary, the primary experimental evidence suggests that the well-known anti-inflammatory and immunomodulatory effects of exercise are at least partly characterized by the ability of chronic exercise to adjust the energetic and biosynthetic demands of lymphocytes in response to physiological and pathological conditions.
